# Molecular epidemiological survey of porcine epidemic diarrhea in some areas of Shandong and genetic evolutionary analysis of S gene

**DOI:** 10.3389/fvets.2022.1015717

**Published:** 2022-10-03

**Authors:** Yesheng Shen, Yudong Yang, Jun Zhao, Ningwei Geng, Kuihao Liu, Yiran Zhao, Fangkun Wang, Sidang Liu, Ning Li, Fanliang Meng, Mengda Liu

**Affiliations:** ^1^School of Animal Science and Technology, Shandong Agricultural University, Tai'an, China; ^2^Huayun (Shandong) Inspection and Quarantine Service Co., Tai'an, China; ^3^Division of Zoonoses Surveillance, China Animal Health and Epidemiology Center, Qingdao, China

**Keywords:** porcine epidemic diarrhea, epidemiological survey, S gene, genetic evolutionary analysis, prevention and control

## Abstract

Responsible for the acute infectious disease porcine epidemic diarrhea (PED), PED virus (PEDV) induces severe diarrhea and high mortality in infected piglets and thus severely harms the productivity and economic efficiency of pig farms. In our study, we aimed to investigate and analyze the recent status and incidence pattern of PEDV infection in some areas of Shandong Province, China. We collected 176 clinical samples of PED from pig farms in different regions of Shandong Province during 2019–2021. PEDV, TGEV, and PORV were detected using RT-PCR. The full-length sequences of positive PEDV S genes were amplified, the sequences were analyzed with MEGA X and DNAStar, and a histopathological examination of typical PEDV-positive cases was performed. RT-PCR revealed positivity rates of 37.5% (66/176) for PEDV, 6.82% (12/176) for transmissible gastroenteritis virus, and 3.98% (7/176) for pig rotavirus. The test results for the years 2019, 2020, and 2021 were counted separately, PEDV positivity rates for the years were 34.88% (15/43), 39.33% (35/89), and 36.36% (16/44), respectively. Histopathological examination revealed atrophied, broken, and detached duodenal and jejunal intestinal villi, as typical of PED, and severe congestion of the intestinal submucosa. Moreover, the results of our study clearly indicate that the G2 subtype is prevalent as the dominant strain of PEDV in Shandong Province, where its rates of morbidity and mortality continue to be high. Based on a systematic investigation and analysis of PEDV's molecular epidemiology across Shandong Province, our results enrich current epidemiological data regarding PEDV and provide some scientific basis for preventing and controlling the disease.

## Introduction

Porcine epidemic diarrhea (PED) is a common viral disease in raising pigs, one with high morbidity, high mortality, and complex causes that are difficult to control (1–4). Among piglets up to 3 months old, PED is especially problematic, with a prevalence of up to 70%. Nevertheless, epidemiological information on PED in large-scale pig farms in recent years in Shandong Province is currently lacking.

PED virus (PEDV), a member of the genus A coronavirus in the Coronaviridae family, is a linear single-stranded, positive-strand RNA virus ~28 kb in length (5). In the virus, ORF4, ORF5, and ORF6 encode four structural proteins—the spine (S) protein, the envelope (E) protein, the membrane (M) protein, and the nucleocapsid (N) protein (6, 7)—among which S proteins, due to their high genetic variability, are often used as markers for phylogenetic analysis and epidemiological investigations (8). Based on the genetic characteristics of the PEDV S gene, it is often divided into two subtypes: G1 and G2 (9–11). Between them, the G2 subtype is the more prevalent genotype of PED in China, whereas the more commonly used vaccine strain CV777 is of the G1 subtype (12, 13).

In our study, we collected 176 clinical samples of PED from pig farms in different regions of In Shandong Province, China during 2019–2021 in order to investigate the prevalence of PEDV there. We performed phylogenetic analysis, comparative nucleotide analysis, and comparative analysis of deduced amino acid sequences with the aim of clarifying the epidemiology of PEDV in Shandong Province.

## Materials and methods

### Sample collection and processing

**Figure 1:**
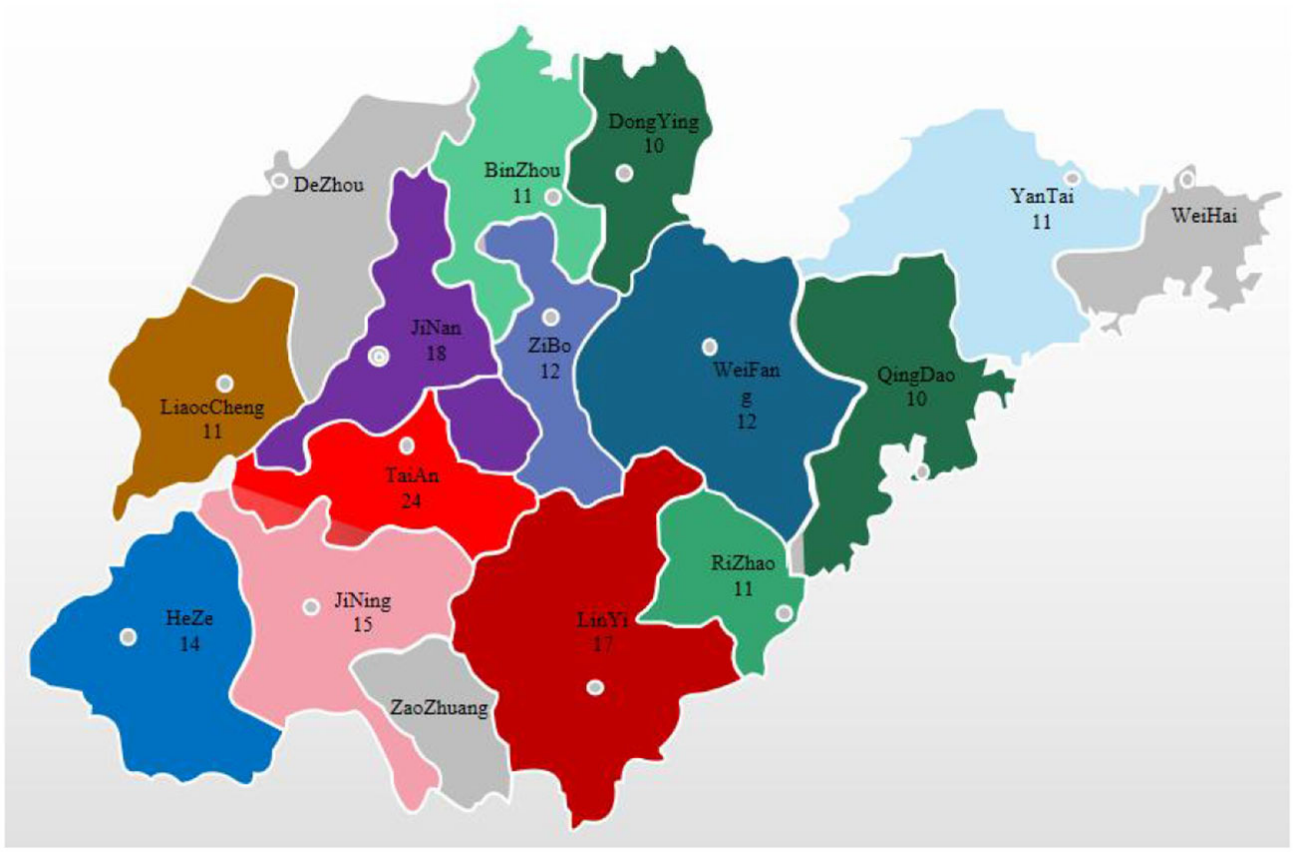
Distribution of sample collection by city in Shandong Province, 2019–2021.

During 2019–2021, we collected 176 intestinal and fecal samples from pigs with diarrheal symptoms on pig farms in localities throughout Shandong Province (Figure 1). The collected samples were ground into tissue homogenates, transferred into 1.5 ml centrifuge tubes, frozen and thawed 3 times, and stored at −80°C for later use.

### RNA extraction and genome amplification

The RNA isolater Total RNA Extraction Reagent R401-01 (Vazyme) was added to the tissue homogenate supernatant, and total RNA was extracted from the intestinal and fecal samples according to the instructions. The primers were synthesized by Sangon; Table 1 shows the sequences of the primers used. Three viruses—PEDV, transmissible gastroenteritis virus (TGEV), and porcine rotavirus (PoRV)—were amplified by RT-PCR using the EasyScript One-Step RT-PCR SuperMix (TransGen Biotech). After 1% agarose gel electrophoresis was performed for 45 min, the results were observed under a gel imaging system. PEDV-positive RNA was reverse-transcribed to cDNA using the ReverTra-Ace First-Strand cDNA Synthesis Kit (Toyobo), after which RT-PCR amplification was performed on PEDV-positive samples using 2 × Phanta Max Master Mix (Vazyme). All steps were performed strictly according to the instructions of the manufacturers.

**Table 1:** The sequences of primers.

**Names**	**Primers (5^′^ → 3^′^)**	**Annealing temperature/°C**	**Fragment size/bp**
PEDV-F PEDV-R	TGTTGTAGGGGTCCTAGACT GGTGACAAGTGAAGCACAGA	54	792
TGEV-F TGEV-R	ATATGCAGTAGAAGACAAT TTAGTTCAAACAAGGAGT	44	1,417
PoRV-F PoRV-R	GGCTTTAAAAGAGAGAATTTC GGTCACATCATACAGTTCTAAC	48	976
PEDV-S1-F PEDV-S1-R	AGATTGCTCTACCTTATACCTG GAAAGAACTAAACCCATTGATA	49	2,192
PEDV-S2-F PEDV-S2-R	AGCCAACTCAAGTGTTCTCAGG AGCCACAGTGTTCAAACCCTT	57	1,691
PEDV-S3-F PEDV-S3-R	TTAATAAAGTGGTTACTAATGGC ATAATAAAGAGCGCATTTTTATA	46	1,823

F represents forward PCR primer; R represents reverse PCR primer.

### Sequencing and phylogenetic analysis

PEDV-positive RT-PCR amplification products were sent to Sangon for sequencing. The sequences were compared using MegAlign in DNAStar and analyzed for nucleotide and deduced amino acid homology with a selection of reference strains entered in the NCBI's database (Table 2). MEGA X was used to construct a genetic evolutionary tree based on the PEDV S gene, while Protean in DNAStar was used to analyze the antigenic epitopes of the isolates.

**Table 2:** Reference strain information.

**Name**	**Distracts**	**GenBank accession no**.
83P-5	Japan	AB548618
AD02	Korea	KC879281
BJ-2012-1	China	JX435299
BrI-87	France	Z25483
CH22-JS	China	JQ979290
GHGD-01	China	JN980698
Chinjμ99	Korea	AY167585
CH-S	China	JN547228
CV777-vaccine	China	JN599150
DR13	Korea	DQ862099
HLJ-2012	China	JX512907
IA2	America	KF468754
JS2008	China	KC210146
KH	Japan	AB548622
MN	America	KF468752
NK	Japan	AB548623
N12-GD2017	China	MK533003.1

### Histopathological observations

The digestive tracts of PEDV-positive pigs were fixed in 10% formalin and removed after 48 h to produce conventional paraffin sections for pathological histological examination using hematoxylin-eosin (HE) staining as a means to observe lesions.

## Results

### Clinical signs and gross lesions

**Figure 2:**
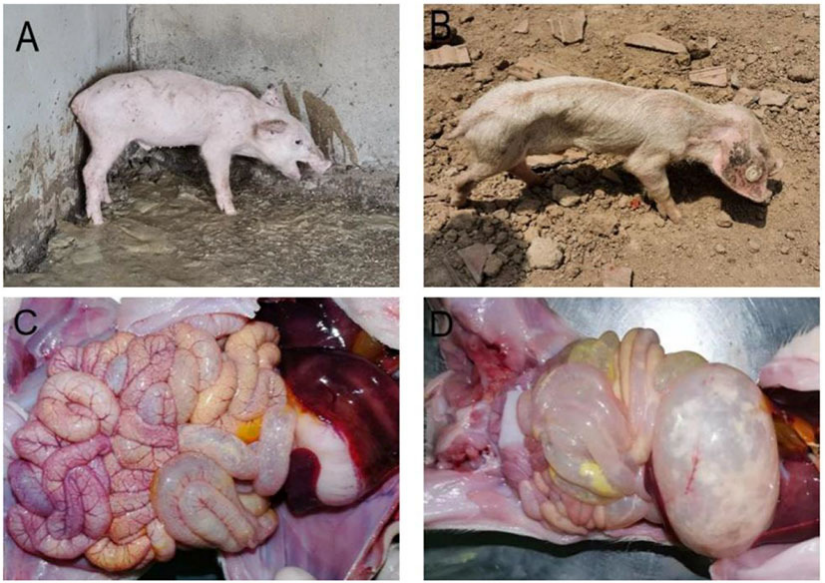
Clinical symptoms and pathological change. **(A)** Diarrhea accompanied by vomiting; **(B)** Dehydration and emaciation; **(C)** Intestinal mucosa and mesenteric hyperemia and bleeding; **(D)** The intestinal wall is clear and filled, and the stomach is dilated.

In practice, severe diarrhea with vomiting (Figure 2A), severe dehydration, and wasting (Figure 2B) can be observed in piglets affected by PED. During autopsies, signs of congestion and hemorrhaging in the intestinal mucosa and mesentery were observed as well (Figure 2C). The walls of the small intestine were thin and transparent, the intestinal lumen was filled with milky or yellow slurry-like contents, and the stomach was clearly dilated and filled with curd-like contents (Figure 2D).

### RT-PCR positive rate of PEDV

**Figure 3:**
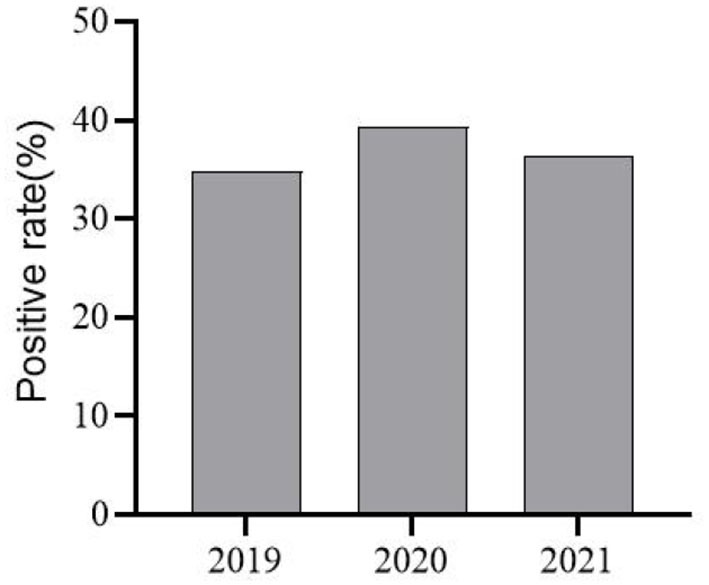
PEDV Infection Positivity in selected areas of Shandong Province from 2019–2021.

RT-PCR revealed positivity rates of 37.5% (66/176) for PEDV, 6.82% (12/176) for TGEV, and 3.98% (7/176) for PoRV. Among them, one case of mixed infection of PEDV and TGEV was found, but no case of mixed infection of PEDV and PoRV was found. When the test results for the years 2019, 2020, and 2021 were counted separately, PEDV positivity rates for the years were 34.88% (15/43), 39.33% (35/89), and 36.36% (16/44), respectively (Figure 3).

### Results of phylogenetic analysis

#### Analysis of genetic variation in the S gene of an endemic strain of PEDV

A total of 12 PEDV S gene sequences were obtained after sequencing (Table 3), all of which were subsequently uploaded to the NCBI's database.

**Table 3:** Information of PEDV positive materials.

**Name**	**Distracts**	**Acquisition time**	**GenBank accession no**.
SDHY_DY	Dongying	12-Dec-20	ON988085
SDHY_ZB	Zibo	20-Nov-20	ON988086
SDHY_YT	Yantai	18-Dec-19	ON988087
SDHY_TA03	Tai'an	10-Jan-21	ON988088
SDHY_TA02	Tai'an	7-Dec-20	ON988089
SDHY_TA01	Tai'an	26-Jan-19	ON988090
SDHY_QD	Qingdao	21-Jan-21	ON988091
SDHY_LW	Ji'nan	20-Nov-19	ON988092
SDHY_LC	Liaocheng	2-Mar-21	ON988093
SDHY_JN02	Ji'ning	24-Nov-20	ON988094
SDHY_JN01	Ji'ning	5-Nov-20	ON988095
SDHY_BZ	Binzhou	2-Mar-21	ON988096

**Figure 4:**
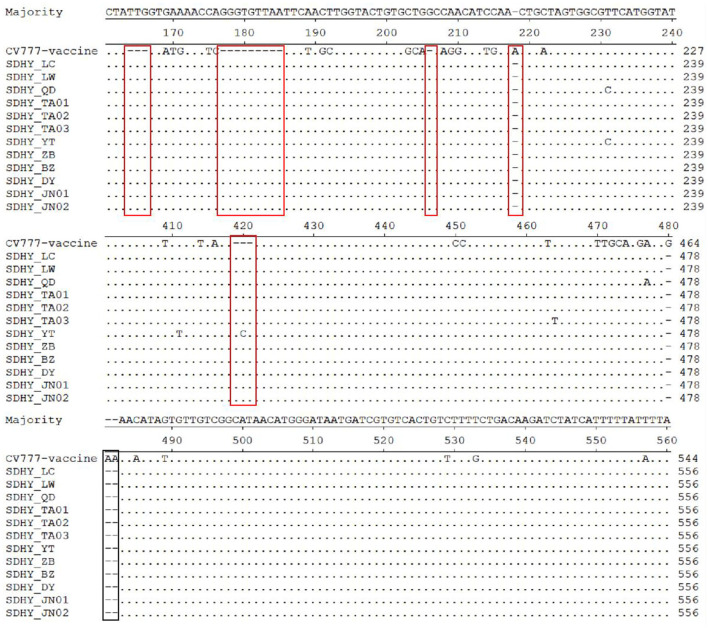
Analysis and comparison of nucleotide of the partial S gene.

A comparative nucleotide analysis of the 12 isolates with the classical vaccine strain CV777 using MegAlign in DNAStar revealed that all isolates had varying degrees of base mutations, deletions, and insertions. In particular, all had three consecutive nt (TTG) insertions at position 164, all had nine consecutive nt (GGGTGTTAA) insertions at position 177, and all had one G insertion at position 206. The SDHY-YT strain had three consecutive nt (ACC) insertions at position 419, whereas the other 11 strains all had AAC insertions. All isolates had an A deletion at position 218 and three consecutive nt (GAA) deletions at positions 480–482 (Figure 4).

**Figure 5:**
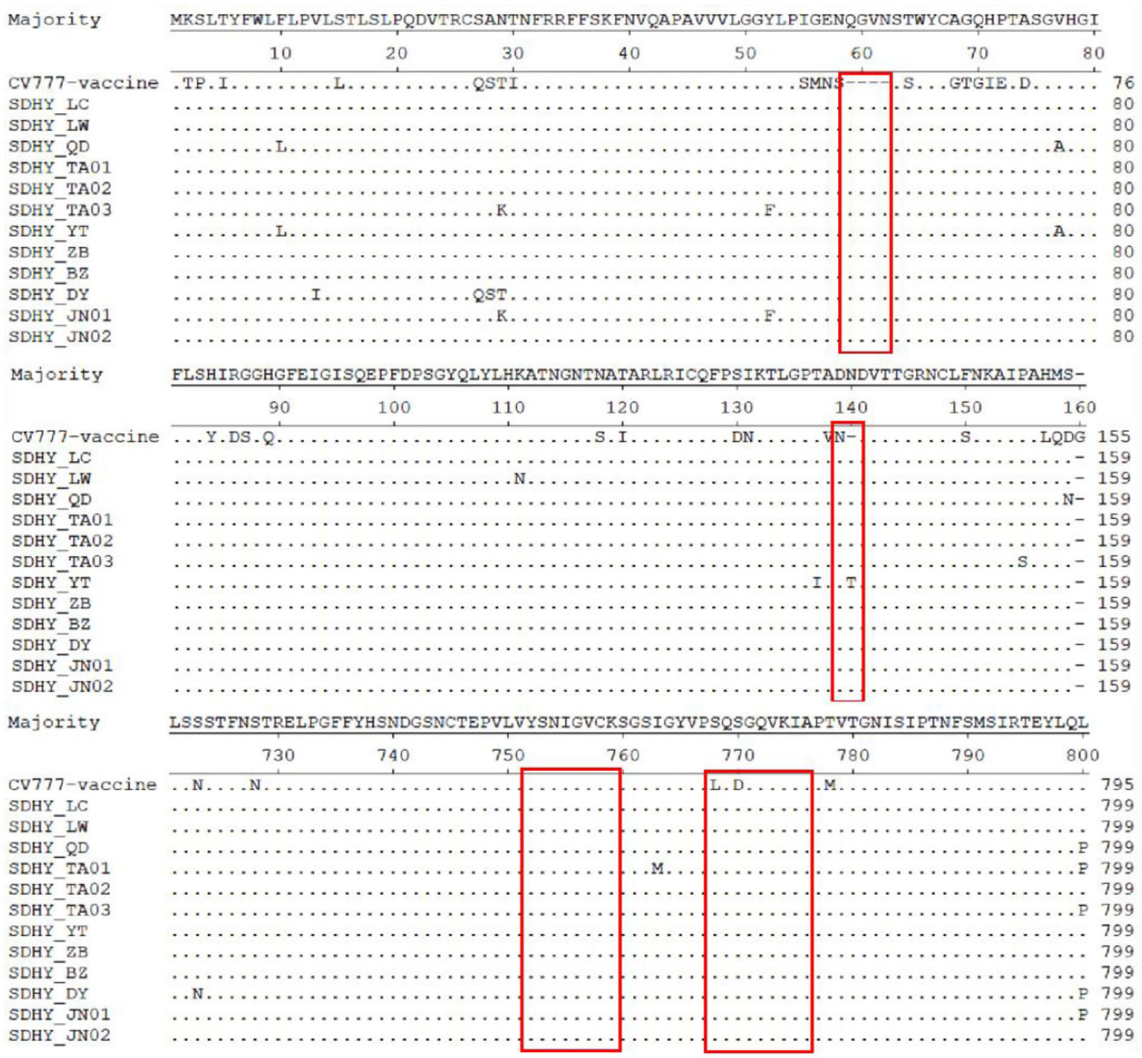
Analysis and comparison of amino acid of PEDV S gene.

A comparison of the deduced amino acid sequences of the 12 isolates with those of CV777 using MegAlign revealed multiple deduced amino acid mutations, deletions, and insertions as well. By contrast, all isolates had four consecutive deduced amino acid (QGVN) insertions at positions 59–62, the SDHY-YT strain had a T insertion at position 140, and all other isolates had an N insertion. All 12 isolates additionally had a G deletion at position 160 (Figure 5).

#### PEDV S gene homology alignment analysis

Analysis using MegAlign in DNAStar revealed that the nucleotide homology among the 12 isolates ranged from 97.2 to 99.9%, while the deduced amino acid homology ranged from 95.9 to 99.7%. Compared with the reference strains, the nucleotide homology was in the range of 93.0–99.1%, while the deduced amino acid homology was in the range of 90.9–99.4%. Compared with the classical vaccine strain CV777, the nucleotide sequence homology was in the range of 93.1–93.9%, while the deduced amino acid sequence homology was in the range of 91.2–93.0% (Table 4).

**Table 4:** Homology analysis of nucleotides and deduced amino acids.

**Name**	**Country**	**Accession number**	**Isolation of viral nt homology**	**Isolation of viral aa homology**
83P-5	Japan	AB548618	93.3–94.1%	91.9–93.6%
AD02	South Korea	KC879281	95.4–96.3%	94.6–9.65%
BJ-2012-1	China	JX435299	97.1–98.1%	96.8–98.5%
BrI-87	France	Z25483	93.2–94.0%	91.7–93.3%
CH22-JS	China	JQ979290	94.6–95.8%	94.1–95.4%
GHGD-01	China	JN980698	96.7–97.4%	96.4–97.8%
Chinju99	South Korea	AY167585	93.1–93.9%	90.9–92.5%
CH-S	China	JN547228	93.1–93.7%	91.8–93.2%
CV777	China	JN599150	93.1–93.9%	91.2–93.0%
DR13	South Korea	DQ862099	93.2–94.0%	91.9–93.5%
HLJ-2012	China	JX512907	97.5–98.7%	97.7–99.0%
IA2	America	KF468754	97.6–98.9%	96.9–99.1%
JS2008	China	KC210146	93.7–94.2%	91.7–93.5%
KH	Japan	AB548622	93.5–94.2%	92.3–93.7%
MN	America	KF468752	97.6–98.6%	96.8–99.0%
NK	Japan	AB548623	93.9–94.8%	92.6–94.0%
N12-GD2017	China	MK533003.1	98.1–99.1%	97.6–99.4%

#### Genetic evolutionary analysis of the PEDV S gene

**Figure 6:**
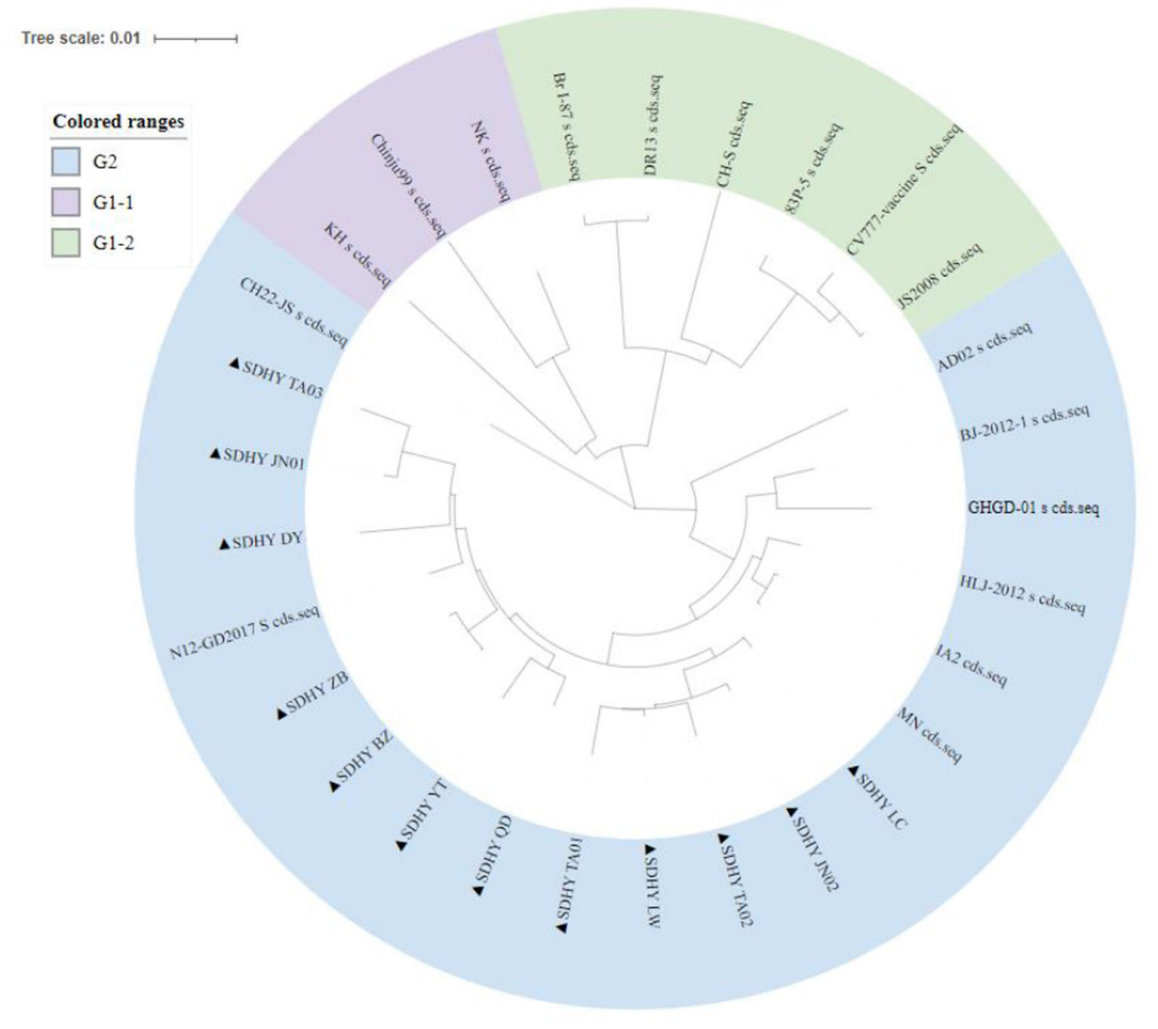
A phylogenetic tree based on the nucleotide construction of the PEDV S gene. “Purple” represents G1-1 subtype, “green” represents G1-2 subtype and “blue” represents G2 subtype. “▴” represents the isolates obtained in this study.

To understand the current genotypes of the dominant strains of PEDV in Shandong Province, we constructed a genetic evolutionary tree (Figure 6) using the neighbor-joining method in MEGA X (14) between the 12 isolates and the reference strains. The results showed that the 12 isolates, all belonging to the G2 genotype, were located in the same branch and in close genetic proximity.

#### Predictive analysis results of PEDV S protein antigen index

**Figure 7:**
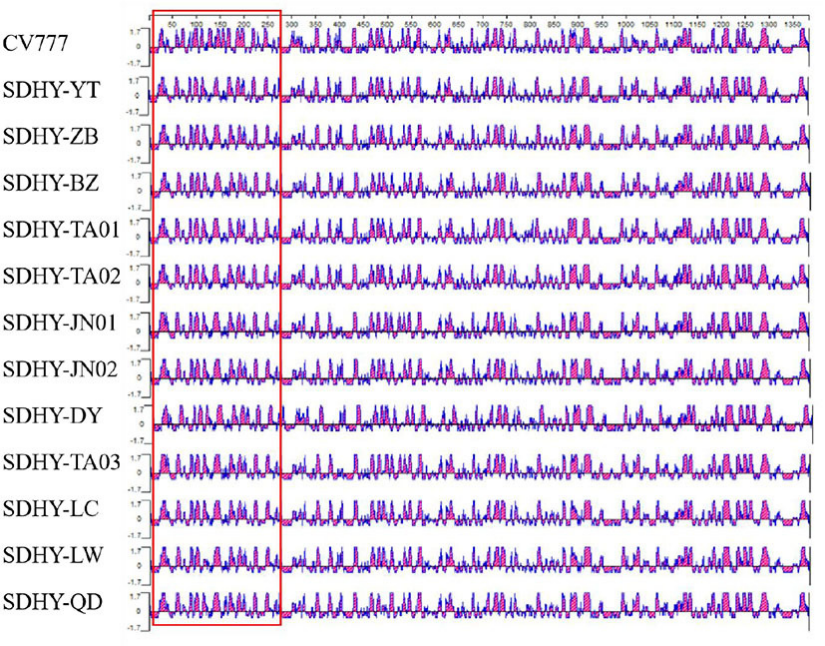
PEDV S protein antigen index prediction.

Next, our antigenic index prediction analysis of the vaccine strain CV777 S protein and the 12 isolated S proteins using the Jameson–Wolf method with Protean in DNAStar revealed that the S protein's overall antigenic index was high, with a significant difference in antigenicity within ~20–280 aa and a less significant difference elsewhere (Figure 7).

### Results of histopathological analysis

**Figure 8:**
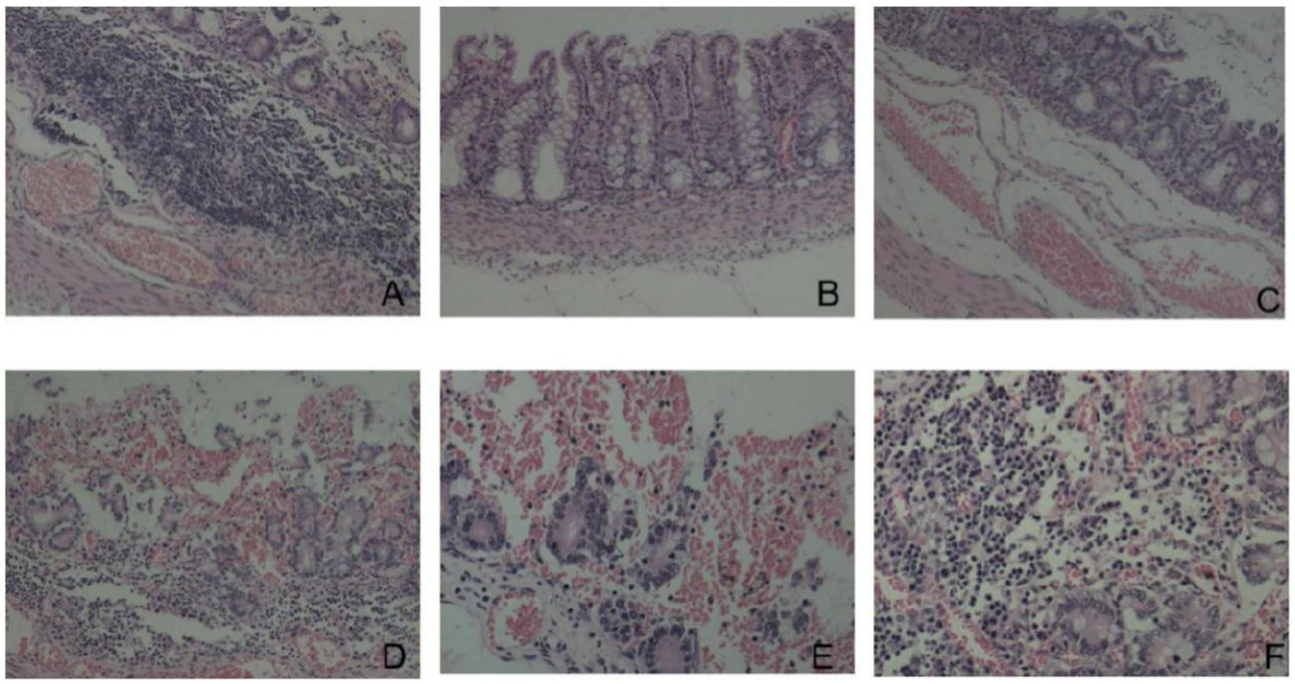
Histopathological changes (HE dyeing). **(A)** Duodenal villi had atrophied and detached, submucosa was congested, ×100; **(B)** increased goblet cells and necrosis of epithelial cells, ×200; **(C)** Necrosis of jejunal mucosal epithelial cells and submucosal hyperemia, ×100; **(D)** rupture and shedding of jejunal villi, ×100; **(E)** jejunum full of necrotic and exfoliated epithelial cells, ×100; **(F)** congestion and inflammatory cell infiltration in the lamina propria of gastric mucosa, ×200.

Histopathological analysis revealed that the duodenal villi had atrophied and detached, and that the submucosa was congested (Figure 8A). The colonic mucosa showed an increased secretion of cupped cells, vacuolation, and the necrosis of epithelial cells (Figure 8B). The jejunal epithelium was necrotic, the submucosa was congested (Figure 8C), the jejunal villi were broken and severely detached (Figure 8D), and the jejunal intestinal canal, filled with necrotic detached epithelial cells, was severely congested and bleeding (Figure 8E). The lamina propria of the gastric mucosa was congested and edematous and showed inflammatory cell infiltration (Figure 8F).

## Discussion

Coping with PED continues to be a daunting challenge for pig farming in China (15). Studies have revealed PEDV positivity rates ranging from 30–45%, with slight differences between northern and southern China (16–19). In our study, PEDV positivity was 37.5% (66/176), TGEV positivity was 6.82% (12/176), and PoRV positivity was 3.98% (7/176); however, PEDV was the primary pathogen responsible for diarrhea in the piglets. By year, PEDV positivity in 2019, 2020, and 2021 was respectively 34.88% (15/43), 39.33% (35/89), and 36.36% (16/44). Overall, PED was shown to be moderately to highly prevalent in some areas of Shandong Province, with relatively stable trends in prevalence. By furnishing those findings, our study has filled gaps in epidemiological data concerning PEDV in Shandong Province in recent years.

In past research, the S gene of PEDV, the primary structural protein gene, has shown significant variation. The S gene sequences of the prevalent strains were systematically analyzed to accurately capture current trends in the strains and identify markers of PEDV variation (20). The results showed that all 12 isolates had varying degrees of base mutations, deletions, and insertions in the S gene, with changes occurring primarily in the N-terminal structural domain (NTD) region of S1 (21). Homology analysis of nucleotides and of deduced amino acid sequences showed that although the 12 isolates were closely related to the Chinese isolate N12-GD2017 from 2017, the American isolates MN and IA2 from 2013, and the Chinese strain HLJ-2012 from 2012, they were more closely related to the classical Chinese vaccine strain CV777, the Japanese strain KH, and the weak Japanese vaccine strain 83P-PEDV. Meanwhile, S1-IDMP were dominated by S1-IDMP S58_S58insQGVN-N135dup-D158_I159del. By contrast, in our study, S1-IDMP were dominated by S1-IDMP S58_S58insQGVN-N135dup-D158_I159del. Taken together, the deduced amino acid sequence mutations in the isolates primarily concentrated in the NTD region of S1. We observed a highly conserved SS2 antigenic epitope sequence (i.e., Y^748^SNIGVCK) within PEDV; however, two AA substitutions (i.e., 764 at L → S and 766 at D → S) occurred at the SS6 antigenic epitope S^764^QSGQVKI. Antigenic index predictions showed a more pronounced difference in antigenicity at 20 aa to 280 aa, with little difference at the remaining positions, which suggests that PEDV S protein changes primarily concentrated in the S1 gene region. In general, the current epidemic strains of PEDV in Shandong Province have high affinity and more obvious mutations in the S1 gene.

Despite the continued high-intensity vaccination of pigs against PED in Shandong Province, the disease can nevertheless occur after multiple vaccinations. In recent years, the incidence of PED in China has shown that the prevalent strains are the main cause of PED, while the incidence and mortality rates have increased year after year (22). In our study, genetic evolutionary analysis showed that all 12 isolates belong to the G2 subtype. However, most of the PEDV vaccines in Shandong Province currently use the CV777 strain of the G1 subtype as their source strain, which may partly explain the occurrence of PED on pig farms in the province. Therefore, we recommend using the more prevalent strain as the source strain for such vaccines, which may yield a more satisfactory immunization effect.

## Conclusion

The results of this study clearly indicate that PEDV is the main pathogen causing diarrheal symptoms in pigs, the G2 subtype is the dominant strain of PEDV in Shandong Province, where its rates of morbidity and mortality continue to be high. At present, the most prevalent strain of PEDV in some areas of the province, however, is only distantly related to the vaccine strain CV777. Using the most prevalent strain for vaccines against PED is therefore recommended. In addition, all 12 PEDV strains S1-IDMP isolated in this study had S58_S58insQGVN-N135dup-D158_I159del-like mutations, which require ongoing attention.

## Data availability statement

The sequences of the isolated strains in this study have been uploaded to GenBanK (accession ON890168 to ON988085-ON988096).

## Ethics statement

All animal protocols and procedures were performed according to the Chinese Regulations of Laboratory Animals and were approved by the Animal Ethics Committee of Shandong Agricultural University.

## Author contributions

YS and YY: writing—original draft preparation. NL, ML, and FM: writing—review and editing. KL and NG: software. YZ, KL, and JZ: investigation. SL: funding acquisition. All authors have read and agreed to the published version of the manuscript.

## Funding

This study was funded by the Shandong Provincial Agricultural Major Application Technology Innovation Project (Establishment and Demonstration of Healthy Pig Breeding Technology Integration and Product Supply Chain Traceability System).

## Conflict of interest

Author FM was employed by company Huayun (Shandong) Inspection and Quarantine Service Co. The remaining authors declare that the research was conducted in the absence of any commercial or financial relationships that could be construed as a potential conflict of interest.

## Publisher's note

All claims expressed in this article are solely those of the authors and do not necessarily represent those of their affiliated organizations, or those of the publisher, the editors and the reviewers. Any product that may be evaluated in this article, or claim that may be made by its manufacturer, is not guaranteed or endorsed by the publisher.

## References

[1] Choudhury B Dastjerdi A Doyle N Frossard JP Steinbach F. From the field to the lab - An European view on the global spread of PEDV. Virus Res. (2016) 226:40–9. 10.1016/j.virusres.2016.09.003 27637348 PMC7114520

[2] Li W Li H Liu Y Pan Y Deng F Song Y. New variants of porcine epidemic diarrhea virus, China, 2011. Emerg Infect Dis. (2012) 18:1350–3. 10.3201/eid1803.120002 22840964 PMC3414035

[3] Shibata I Tsuda T Mori M Ono M Sueyoshi M Uruno K. Isolation of porcine epidemic diarrhea virus in porcine cell cultures and experimental infection of pigs of different ages. Vet Microbiol. (2000) 72:173–82. 10.1016/S0378-1135(99)00199-6 10727829 PMC7117361

[4] Wang Q Vlasova AN Kenney SP Saif LJ. Emerging and re-emerging coronaviruses in pigs. Curr Opin Virol. (2019) 34:39–49. 10.1016/j.coviro.2018.12.001 30654269 PMC7102852

[5] Pensaert MB de Bouck P. A new coronavirus-like particle associated with diarrhea in swine. Arch Virol. (1978) 58:243–7. 10.1007/BF01317606 83132 PMC7086830

[6] Chen P Wang K Hou Y Li H Li X Yu L. Genetic evolution analysis and pathogenicity assessment of porcine epidemic diarrhea virus strains circulating in part of China during 2011-2017. Infect Genet Evol. (2019) 69:153–65. 10.1016/j.meegid.2019.01.022 30677534 PMC7106134

[7] Jung K Saif LJ Wang Q. Porcine epidemic diarrhea virus (PEDV): an update on etiology, transmission, pathogenesis, and prevention and control. Virus Res. (2020) 286:198045. 10.1016/j.virusres.2020.198045 32502552 PMC7266596

[8] Li N Liu J Qi J Hao F Xu L Guo K. Genetic diversity and prevalence of porcine circovirus type 2 in China during 2000-2019. Front Vet Sci. (2021) 8:788172. 10.3389/fvets.2021.788172 34977219 PMC8717868

[9] Antas M Olech M Szczotka-Bochniarz A. Molecular characterization of porcine epidemic diarrhoea virus (PEDV) in Poland reveals the presence of swine enteric coronavirus (SeCoV) sequence in S gene. PLoS ONE. (2021) 16:e0258318. 10.1371/journal.pone.0258318 34714840 PMC8555794

[10] Chen J Liu X Shi D Shi H Zhang X Li C. Detection and molecular diversity of spike gene of porcine epidemic diarrhea virus in China. Viruses. (2013) 5:2601–13. 10.3390/v5102601 24153062 PMC3814607

[11] Dortmans J Li W van der Wolf PJ Buter GJ Franssen PJM van Schaik G. Porcine epidemic diarrhea virus (PEDV) introduction into a naive Dutch pig population in 2014. Vet Microbiol. (2018) 221:13–8. 10.1016/j.vetmic.2018.05.014 29981699 PMC7117506

[12] Bi J Zeng S Xiao S Chen H Fang L. Complete genome sequence of porcine epidemic diarrhea virus strain AJ1102 isolated from a suckling piglet with acute diarrhea in China. J Virol. (2012) 86:10910–1. 10.1128/JVI.01919-12 22966198 PMC3457323

[13] Shi W Hao H Li M Niu J Hu Y Zhao X. Expression and purification of a PEDV-neutralizing antibody and its functional verification. Viruses. (2021) 13:472. 10.3390/v13030472 33809239 PMC7999980

[14] Tamura K Stecher G Peterson D Filipski A Kumar S. MEGA6: molecular evolutionary genetics analysis version 6.0. Mol Biol Evol. (2013) 30:2725–9. 10.1093/molbev/mst197 24132122 PMC3840312

[15] Turlewicz-Podbielska H Pomorska-Mól M. Porcine coronaviruses: overview of the state of the art. Virol Sin. (2021) 36:833–51. 10.1007/s12250-021-00364-0 33723809 PMC7959302

[16] Chen X Zhang XX Li C Wang H Wang H Meng XZ. Epidemiology of porcine epidemic diarrhea virus among Chinese pig populations: a meta-analysis. Microb Pathog. (2019) 129:43–9. 10.1016/j.micpath.2019.01.017 30682525

[17] Lee DU Kwon T Je SH Yoo SJ Seo SW Sunwoo SY. Wild boars harboring porcine epidemic diarrhea virus (PEDV) may play an important role as a PEDV reservoir. Vet Microbiol. (2016) 192:90–4. 10.1016/j.vetmic.2016.07.003 27527769 PMC7117357

[18] Mai TN Yamazaki W Bui TP Nguyen VG Le Huynh TM Mitoma S. A descriptive survey of porcine epidemic diarrhea in pig populations in northern Vietnam. Trop Anim Health Prod. (2020) 52:3781–8. 10.1007/s11250-020-02416-1 33011908 PMC7532947

[19] Reveles-Félix S Carreón-Nápoles R Mendoza-Elvira S Quintero-Ramírez V García-Sánchez J Martínez-Bautista R. Emerging strains of porcine epidemic diarrhoea virus (PEDv) in Mexico. Transbound Emerg Dis. (2020) 67:1035–41. 10.1111/tbed.13426 31733175 PMC7159366

[20] Su M Li C Qi S Yang D Jiang N Yin B. A molecular epidemiological investigation of PEDV in China: characterization of co-infection and genetic diversity of S1-based genes. Transbound Emerg Dis. (2020) 67:1129–40. 10.1111/tbed.13439 31785090 PMC7233288

[21] Wang E. Molecular epidemiological investigation and virus isolation and identification of S1 gene of porcine epidemic diarrhea virus in China from 2015 to 2016 (Master), Heilongjiang Bayi Agricultural University (2018).

[22] Gerdts V Zakhartchouk A. Vaccines for porcine epidemic diarrhea virus and other swine coronaviruses. Vet Microbiol. (2017) 206:45–51. 10.1016/j.vetmic.2016.11.029 27964998 PMC7117160

